# Tetracalcium Phosphate/Monetite/Calcium Sulfate Hemihydrate Biocement for Alveolar Bone Augmentation After Tooth Extraction in Pig Mandible

**DOI:** 10.3390/bioengineering11111057

**Published:** 2024-10-24

**Authors:** Katarína Vdoviaková, Lenka Krešáková, Filip Humeník, Ján Danko, Kristína Čurgali, Andrej Jenča, Andrej Jenča, Adriána Petrášová, Janka Jenčová, Marko Vrzgula, Mária Giretová, Radoslava Štulajterová, Ľubomír Medvecký

**Affiliations:** 1 Department of Morphological Disciplines, University of Veterinary Medicine and Pharmacy in Košice, Komenskeho 73, 041 81 Košice, Slovakia; katarina.vdoviakova@uvlf.sk (K.V.); lenka.kresakova@uvlf.sk (L.K.); filip.humenik@uvlf.sk (F.H.); jan.danko@uvlf.sk (J.D.); 2 Department of Histology and Embryology, Faculty of Medicine, Pavol Jozef Šafárik University in Košice, Šrobárova 2, 041 83 Košice, Slovakia; kristina.curgali@upjs.sk; 3 Solmea s.r.o., Bačíkova 7, 040 01 Košice, Slovakia; andrej.jenca@upjs.sk (A.J.); andrej.jenca1@upjs.sk (A.J.J.); adriana.petrasova@upjs.sk (A.P.); janka.jencova@upjs.sk (J.J.); 4 Department of Anatomy, Faculty of Medicine, Pavol Jozef Šafárik University in Košice, Šrobárova 2, 041 83 Košice, Slovakia; marko.vrzgula@upjs.sk; 5 Division of Functional and Hybrid Materials, Institute of Materials Research of SAS, Watsonova 47, 040 01 Košice, Slovakia; rstulajterova@saske.sk (R.Š.); lmedvecky@saske.sk (Ľ.M.)

**Keywords:** composites, cement, socket preservation, healing, pig animal model

## Abstract

A tetracalcium phosphate/monetite/calcium sulfate hemihydrate powder cement mixture (CAS) in the form of a paste was used as a socket preservative to prevent alveolar ridge resorption after the extraction of the second premolar tooth in the mandible of a pig model. During the post-surgery period, the animals were monitored daily. No signs of inflammation, infection, or other complications were observed in the defect site for six months. Six months after surgery, the mandible defects in pigs were evaluated using macroscopic, histological, and radiological techniques. Treatment of the extraction sites with CAS biocement resulted in the uniform filling of the defects with alveolar bone tissue, characterized by a relatively smooth and homogeneous gum surface with no visible biocement residues. The formation of new bone tissue with osteoblasts, osteocytes, and mineralized matrices was confirmed. The results showed the similar morphology, thickness, and complete integration of the newly formed bone with the surrounding tissue. CAS biocement could be an effective material to prevent alveolar bone reduction as well as soft tissue loss and could support dental implant placement with long-term functionality.

## 1. Introduction

Ridge preservation is a procedure that reduces bone and soft tissue loss after tooth extraction. Tooth extraction in humans is mostly induced by deep caries, periodontitis, endodontic lesions, or trauma and induces a series of biological processes. These processes begin with hemostasis. Following the formation of a blood clot, granulation tissue begins to infiltrate the clot from the base of the socket; seven days after extraction, the granulation tissue completely replaces the clot and uncalcified bone tissue (osteoid) forms at the base of the socket. Osteoid begins to mineralize after 4 weeks post extraction. This is followed by complete reepithelialization, which covers the socket by six–eight weeks post extraction [1,2,3,4].

The most significant and critical outcome after tooth extraction is the resorption and remodeling of the alveolar bone, leading to an irreversible reduction in the height and width of the alveolar ridge [2,3,5,6,7]. The greatest alveolar bone volume reduction following an extraction occurs in the first 6 months to 2 years [8] and continues at a mean 0.5–1% per year for life due to the loss of the periodontal ligament and a lack of mechanical stimulation [9,10]. Major changes take place in the 12 months following an extraction with an average 50 percent reduction in the width of the alveolar ridge. Two-thirds of this reduction occur within the first three months [2,11]. Clinical results indicate that alveolar bone loss ranges from 29% to 63% horizontally and 11–22% vertically at 6 months after tooth extraction—mean horizontal reduction in ridge width ~3.8 mm, mean vertical reduction in ridge height ~1.24 mm [12], and alveolar bone loss occur mainly at the buccal area. The volume loss of bone mass after 2 years can reach ~60% in the absence of grafting [13]. Alveolar ridge resorption causes serious clinical problems because an appropriate alveolar ridge size is required for proper implant placement, and thus proper long-term implant function [14]. Several studies have reported that alveolar ridge preservation via socket augmentation with bone grafts effectively prevents physiologic bone loss, soft and hard tissue collapse, and can minimize the need for future augmentation procedures [15]. Socket preservation procedures are designed to minimize alveolar ridge resorption and maximize bone formation within the extraction socket. The goal is to maintain a stable ridge volume to optimize functional and aesthetic long-term results. Alveolar ridge reconstruction can be performed using several types of bone grafts, including autogenous (bone of the same individual), allogenous (bone of the same species, but not of the same individual), xenogenous (material of biologic origin but of different animal species, algae, proteins), and alloplastic (material of synthetic origin such as calcium phosphates, bioglass, ceramics, and polymers) [5,16]. Research teams have evaluated also the influence of platelet-rich plasma on the healing of extraction sockets [17,18,19]. Alveolar ridge preservation in human clinical studies has employed combinations of stem cells, growth factors, and platelet-rich fibrin in various combinations with collagen, bone allografts, and calcium phosphates [20].

Synthetic bone grafting materials such as hydroxyapatite, α- or β-tricalcium phosphates, calcium sulphate, polymers, and composites have been developed to avoid certain limitations of allografts, autografts, and xenografts (risks of disease transmission and immune responses) [14,21]. Studies have shown that nano-sized ceramics are a promising class of bone substitutes in terms of their improved osteointegration. Calcium phosphate-based cements (CPC) with excellent biocompatibility, osteoconduction, and bioresorption are used for the treatment of bone defects. Tetracalcium phosphate/monetite powder mixtures (TTCPM) are characterized by fast setting and the conversion of cement components to calcium-deficient hydroxyapatite (CDHA) after being mixed with a liquid component [22]. Their disadvantages could include a slower rate of resorption in vivo, a relatively rapid change of pH to a strong basic region after the application of the cement paste as well as insufficient osteoinduction after the defect treatment, but all these parameters can be modified by changing the composition of cement mixtures.

Calcium sulfate (CS) displays excellent biocompatibility but it is resorbed too rapidly (due to relative high solubility) in relation to the new bone tissue growth. It possesses good osteoconductive and osteointegration properties which predispose it to multiple medical applications, such as bone defect reconstruction, drug delivery, guided tissue regeneration, endodontic surgery, sinus augmentation, and also alveolar ridge preservation. It is a cheap material, commonly available and easily processed [23,24]. The calcium ions released during its dissolution activate platelets to release bone morphogenetic proteins and platelet-derived growth factors that stimulate the proliferation and osteogenic differentiation of mesenchymal stem cells. CS promotes bone growth and enhances angiogenesis. Therefore, it is considered a bioactive graft material [13]. CS was fully resorbed within 3 months in human fresh extraction sockets and improved bone healing [25].

Extracts from TTCPM/CSH composite cements were demonstrated to induce the overexpression of alkaline phosphatase, osteopontin, osteonectin, and collagen I osteogenic genes in vitro [26].

The objective of this study was to investigate the potential of tetracalcium phosphate/monetite/calcium sulfate hemihydrate biocement (CAS) as a suitable candidate for socket preservation to prevent alveolar ridge resorption following tooth extraction in a pig model. The post-extraction sites filled with CAS cement mixture were evaluated macroscopically, histologically, and radiologically after 6 months of healing to assess the quality of the healing process in terms of new bone formation and its interface with the adjacent bone.

## 2. Materials and Methods

### 2.1. Preparation of TTCP/Monetite/Calcium Sulfhate Hemihydrate Cement

CAS cement powder mixtures were prepared according to the method described in ref. [26]. Briefly, the TTCPM/CSH (CSH—calcium sulfate hemihydrate) powder cement mixture was synthesized using an in situ reaction of TTCP (tetracalcium phosphate) with an orthophosphoric acid (86% analytical grade, Merck, Darmstadt, Germany)/H_2_SO_4_ (96%, analytical grade, Merck) mixture in 80 *v/v*% ethanol (reaction solution) using a planetary ball mill with agate balls and a vessel for 30 min. The orthophosphoric acid was added in such an amount to have the Ca/P mole ratio in the cements close to 1.67 (designated C cement, free of CSH) and sulfuric acid was added in the amount of 5 wt% of CSH in the final designated cement. The cement pastes were prepared by mixing the powder mixtures with 2% NaH_2_PO_4_ (as a liquid component) at a powder/liquid (P/L) ratio = 2. The final cement paste was prepared just before application to the alveolar ridge defect immediately following premolar P2 extraction by mixing the sterile CAS cement with 2% NaH_2_PO_4_ (as liquid component, sterile solution).

### 2.2. In Vitro Cytotoxicity Testing of CAS Cement Extracts

According to ISO 10993-12:2012 [27], extracts of C and CAS cements were prepared (0.2 g/mL culture medium) and soaked overnight. MC3T3E1 preosteoblasts (ATCC CRL-2593, Manassas, VA, USA) were seeded in the wells of a 96-well culture plate at a density of 10^4^ cells/100 μL culture medium/well and cultured overnight. The culture medium used for extraction and cultivation was EMEM (Minimum Essential Medium Eagle) with 10% FBS and 1% ATB-ATM (all from Sigma, Saint-Louis, MO, USA). After 24 h, the extracts were sterile filtered, the culture medium was discarded and replaced with the extracts and incubated for the next 24 h. The viability of cells in extracts was examined with an MTS test (Cell Titer Aqueous One Solution Cell Proliferation Assay, Promega, Madison, WI, USA) according to ISO 10993–5 [28].

### 2.3. Experimental Animal Model

The experiment was performed in accordance with the guidelines of the State Veterinary and Food Administration of the Slovak Republic (study No. 4650/17-221). Eight adult Large White female pigs (breeding farm PD Agro, Michalovce, Slovak Republic) with an average weight of 258.9 kg ± 3.57 kg (mean ± standard deviation) were included in the study for the examination of the alveolar bone regenerative potential in the pig mandible. The first, experimental group (n = 4) consisted of animals that were implanted with calcium phosphate biomaterial into the alveolar bone of the left mandible after the extraction of the second premolar. In the second, control group (n = 4), defects were spontaneously healed after the extraction of the same tooth on the left side. Animals were housed in the Swine clinic of the University of Veterinary Medicine and Pharmacy in Košice (Košice, Slovakia) and checked daily by qualified animal personnel. All pigs were fed with a standard diet and were given free access to water during the in vivo testing.

#### 2.3.1. Premedication and General Anesthesia

The premedication of the animals composed of the mixture of teletamine 50 mg/mL and zolazepam 50 mg/mL (ZOLETIL 100 Vet., Virbac, Nice, France), ketamine 2.5 mL (Ketamidor 100 mg/mL, VetViva Richter GmbH, Wels, Austria), xylazine 2.5 mL (Xylased 100 mg/mL, Bioveta SK spol., s.r.o., Nitra, Slovak Republic), butorphanol 1.5 mL/100 kg (Butomidor 10 mg/mL, VetViva Richter GmbH, Wels, Austria), and was administered intramuscularly. We introduced a cannula into the left lateral auricular vein (v. auricularis lateralis sinistra), with which we applied the anesthetic during the operation, based on the monitored vital functions of the animal.

#### 2.3.2. Tooth Extraction and Filling of the Alveolar Bone Defect

**Figure 1:**
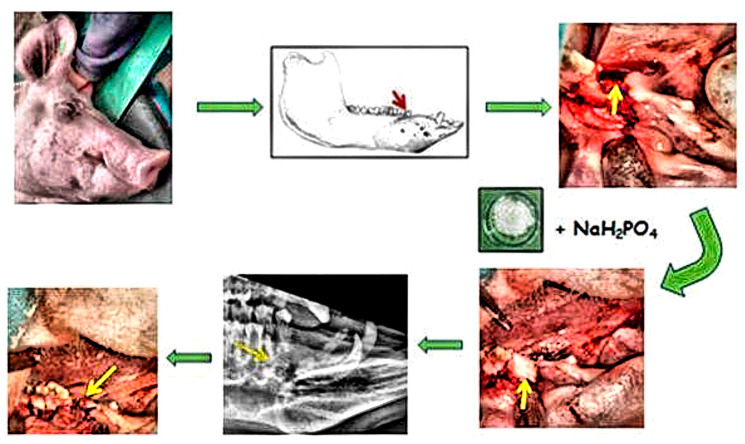
Schematic diagram of the alveolar bone defect study design. The surgery was performed on the left side of the mandible at the level of the second premolar. The created bone defect was filled by CAS biocement paste. A control X-ray examination was performed to confirm the correct filling of the alveolar bone defect (red and yellow arrows indicate the implantation site).

Using an intra-oral approach with mouth gags, unilateral defects of the alveolar bone at the level of the second premolar in the mandible were created. For experimental purposes, the left, second mandibular premolar of the pigs was extracted by means of pliers. After the tooth was extracted, the created socket was enlarged (diameter—10 mm, depth—10 mm). In the first group, the sterile CAS in the form of a paste was applied to the alveolar bone defect on the left side of the mandible at the level of the second premolar (Figure 1). The alveolar bone defect was left empty for spontaneous healing in the second group. Note that the bleeding did not cause complications in filling the resulting defect but can stimulate the defect’s healing. After the surgical procedure, the wound was closed in layers with resorbable sutures. The conventional radiographs of the lateral view porcine mandible were used to confirm the complete filling of the defect by biocement paste post-surgery (Figure 1).

#### 2.3.3. Post-Operative Care

During post-operative care, animals were fed with a liquid feed for the first week after surgery then the standard diet of the animals was the same as the preoperative period. All animals were given free access to water during their convalescence. The animals were monitored daily in the post-operative period, until the end of the experiment. The following parameters were evaluated: state of general health, body temperature, fecal consistency, surgical wound, signs of infection or any other complications. The post-surgery period consisted of the application of antibiotic prophylaxis with repeated doses of the broad-spectrum antibiotic oxytetracycline dihydrate 1 mL/10 kg body weight (Alamycin LA a.u.v., Norbrook, Newry, UK), intramuscularly once every other day for 7 days, and repeated injections of the non-steroidal anti-inflammatory drug flunixin meglumine 2.2 mg/kg body weight (Flunixin a.u.v., Norbrook, Newry, UK), intramuscularly once a day for 7 days.

#### 2.3.4. Animal Euthanasia and Specimen Retrieval

Euthanasia of the animals was performed 6 months after CAS application. After the sedation of the animals with azaperone 2 mg/kg body weight (Stresnil 40 mg/mL Janssen Pharmaceutica, Beerse, Belgium) intramuscularly, euthanasia was performed intravenously with thiopental 90 mg/kg body weight (Thiopental VUAB 1.0 g, VUAB, Pharma a.s., Roztoky u Prahy, Czech Republic). The condition of the gums and surgical wounds was evaluated after removing the skin and muscles, and the newly formed bone was subsequently revealed. The bone surface was examined macroscopically, digitally photographed, and harvested for documentation. The healed mandible bone defect sites were analyzed in more detail histologically and by radiographic imaging.

### 2.4. Assessment of Alveolar Bone Tissue Regeneration

#### 2.4.1. Macroscopic Evaluation

After the animal was euthanized, its mandible bone defect was evaluated in situ as was the status of the gum and surgical wound. The macroscopic evaluation of each wound included the color and the signs of inflammation, infection, bleeding, and the presence of transudate or other damage of the tissue after the extraction of the tooth. The bone tissue at the site of the healed defect was subjected to a macroscopic examination, where the color, surface, filling of the defect, and the integration of the newly formed bone tissue with the surrounding bone were monitored.

#### 2.4.2. Histological Examination

The specimens of the mandible were obtained by collecting a 1.0 cm long, 1.0 cm wide, and 1.0 cm thick bone block using a bone saw, fixed in neutral formalin for one week. Bone specimens were decalcified in Chelaton during a three-week period and washed in sterile distilled water for several hours following decalcification. After this procedure, bone tissues were dehydrated in alcohol and embedded in Paraplast. Embedded specimens were serially sectioned at a 7 µm thickness in the sagittal plane, mounted on slides, stained with hematoxylin–eosin, and prepared for histological analysis.

#### 2.4.3. Radiological Examination

Six months after the surgical procedure, the conventional radiographic examination (X-ray) and computer tomography (CT) of the mandible were carried out to evaluate the regeneration of the alveolar bone tissue at the level of the second premolar tooth. The radiographs in the latero-lateral position of the porcine head using an X-ray instrument (Philips Digital Diagnost, Delft, Netherlands) were recorded. The computer tomography scans of the porcine head we performed in the axial and sagittal planes (using the instrument Philips Brilliance 40-slice CT, Netherlands), where the new alveolar bone tissue formation of the mandible at the level of the second premolar was evaluated.

## 3. Results

### 3.1. Mechanical and Physico-Chemical Properties of CAS Cement

The properties of CAS cements were described in more detail in ref. [26]. Briefly, the final powder cement mixture was composed of homogeneously distributed tetracalcium phosphate microparticles of monetite and calcium sulfate hemihydrate nanoparticles. The setting time and compressive strength of the hardened cement were ~5 min and 31 MPa, respectively. The CAS cement was characterized by 56% porosity and the rapid release of calcium ions due to the dissolution of soluble CSH in physiological solution 

The pH of simulated body fluid solution during the soaking of the CAS was close to the physiological value (~7.5). The final product of cement transformation was nanocrystalline calcium-deficient hydroxyapatite without the remains of calcium sulfates.

### 3.2. In Vitro Cytotoxicity Testing

**Figure 2:**
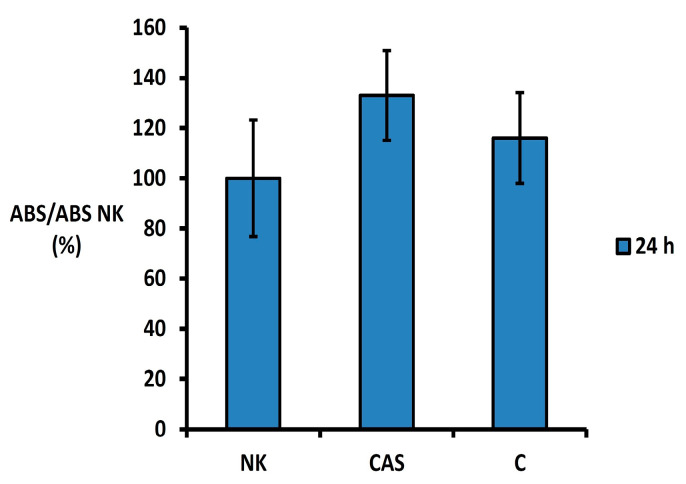
Viability of osteoblasts in cement extracts. (ABS-absorbance, NK-negative control, CAS-tetracalcium phosphate/monetite/calcium sulfate cement mixture, C-TTCP/monetite).

The MTS proliferation test showed no cytotoxic effect of the CAS cement extract 24 h after extraction on the osteoblastic cell line (Figure 2). The absorbance of formazan in wells with CAS extract in relation to the absorbance of the negative control (cells cultured in extract-free culture medium) exceeded 133% but was not statistically different (*p* > 0.43) from the negative control or C cement (without CAS).

### 3.3. Experimental Animal Model and Macroscopic Evaluation of Treated Defect

The overall health of all animals was satisfactory throughout the post-operative period. In two animals, swelling was observed in the area of the surgery wound two days after surgery, but it disappeared without therapy. In the post-operative period, wound dehiscence, macroscopic symptoms of inflammation, infection or other complications were not detected at the defect site. All animals used in our experiment tolerated the implantation of CAS biocement paste. The pigs did not lose body weight during the healing period.

**Figure 3:**
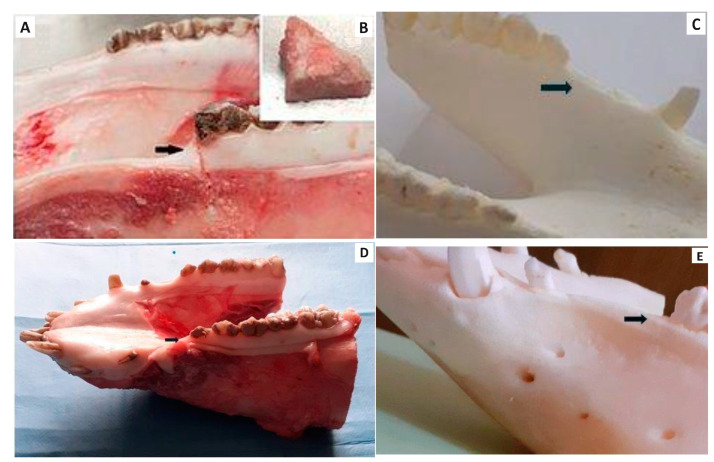
Macroscopic assessment of the treated alveolar bone defect using CAS, the black arrow shows the place of alveolar bone defect on (**A**) the fresh material, (**B**) the cross section of the new bone tissue, (**C**) the bone tissue after maceration). Macroscopic assessment of the bone defect after spontaneous healing, on the fresh mandible (**D**) and after maceration (**E**).

The study design is clearly displayed in Figure 1. Macroscopic evaluation confirmed that the mandible wound at the level of the second premolar was well healed in all experimental animals in the group with the biomaterial CAS, after six months of healing. Macroscopic evaluation of bone tissue showed that the new alveolar bone tissue at the level of the second premolar was ivory white with good integration with the adjacent native bone (Figure 3A). A longitudinal incision of the new bone confirmed that the healing process led to a uniform filling of the defect with new alveolar bone tissue characterized by a relatively smooth and homogeneous surface (Figure 3B). No residue of calcium phosphate biocement (Figure 3C) was detected at the site of the bone defect six months after implantation. The macroscopic evaluation of the mandible bone defects after spontaneous healing also revealed similar results (Figure 3D,E).

### 3.4. Histological Evaluation

**Figure 4:**
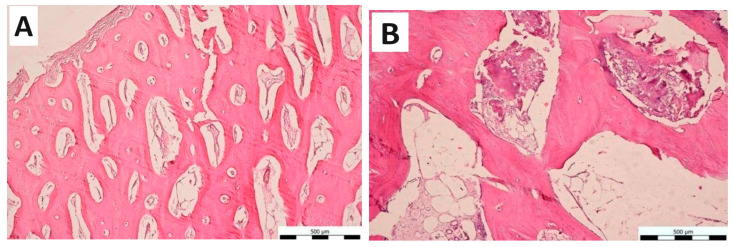
Representative microphotograph of a bone defect filled with CAS material. A Periosteum was observed on the surface of new bone tissue and secondary bone tissue predominated over primary bone tissue. The bone tissue was regenerated more efficiently and its overall appearance fully resembled the original bone. (Mg. 10 × 10), (**A**) Representative microphotograph of the bone defect in the control group. Primary woven bone tissue with slightly hypertrophic lacunae prevailed. The regeneration was incomplete; significant histomorphological changes can be observed in the bone tissue. (Mg. 10 × 10), (**B**).

CAS filling material was excellently tolerated by adjacent bone tissue. Six months after the creation of the bone defect, a complete resorption of the used biomaterial was observed with no visible boundaries between the original intact bone tissue and the new bone tissue. The biocement was perfectly tolerated and the overall histological picture did not show any inflammatory reaction or the presence of inflammatory cells within the new tissue, which indicated the excellent tolerance and biocompatibility of the filling material. From the histomorphological point of view, we observed the dominance of secondary lamellar bone tissue, with fully developed lamellar systems. Osteons with narrow regularly shaped Haversian canals and interstitial lamellae were identified in compact bone tissue. Moreover, the bone trabeculae were developed in spongy bone tissue. Primary woven bone tissue was scarcely present, which demonstrates the ongoing remodeling of immature bone tissue into mature lamellar bone tissue. Osteoprogenitor cells commonly with osteoblasts, osteocytes, and osteoclasts were present on the surface of the bone tissue. Osteocyte lacunae were of normal size without serious morphological and structural changes. The outer and inner surface of the bone was covered by the periosteum and well-developed endosteum, respectively. The cavities between the trabeculae of lamellar bone tissue were filled with yellow bone marrow (Figure 4A). Compared to the control group (defect not treated with cement), the bone tissue in the treated defect regenerated more efficiently, and its microscopic structure and overall appearance resembled undamaged physiological bone. In the control group, healing was incomplete. Cavities in the bone tissue were extensive, and regeneration was not complete even six months after surgery. In addition, the primary woven bone tissue with slightly hypertrophic lacunae was dominant. Significant calcifications were observed on the periphery of the bone. In the overall histomorphological image, the sites of the bone defects with significant histopathological changes were easily distinguishable (Figure 4B). The control sample showed greater pathological changes in the microarchitecture of new bone tissue in comparison with treated bone defects.

### 3.5. Radiological Evaluation

**Figure 5:**
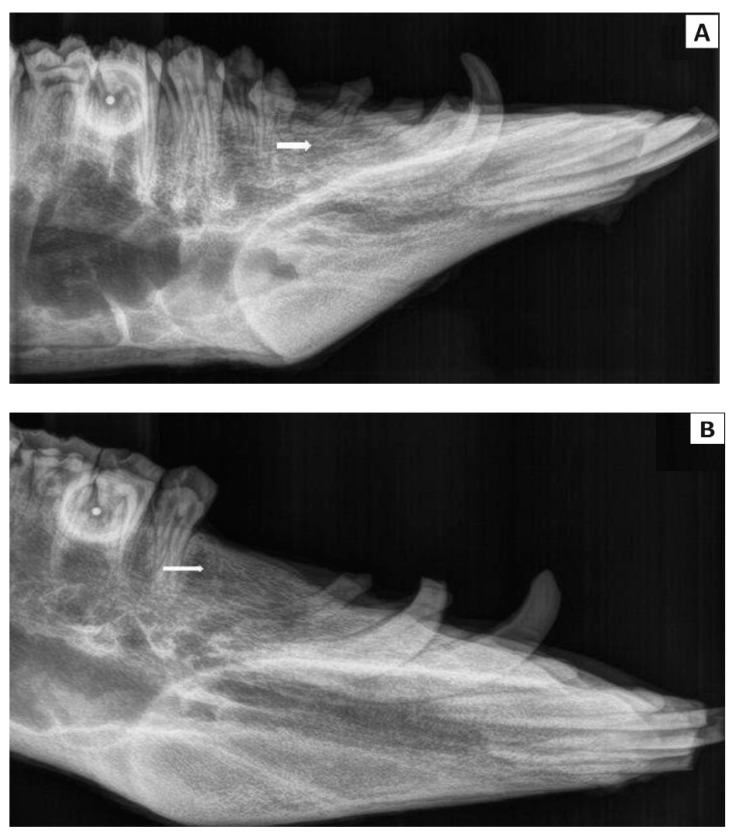
Standard radiographs with required spatial resolution of the left lower jaw at the level of the second premolar of the evaluated area of the defect in the treated part of the alveolar bone, in the *experimental group*, the mandible defects were filled with newly formed bone tissue, which was completely integrated with the surrounding healthy bone (**A**, white arrow), and in the *control group*, the new bone tissue was not fully integrated with adjacent bone tissue (**B**, white arrow).

**Figure 6:**
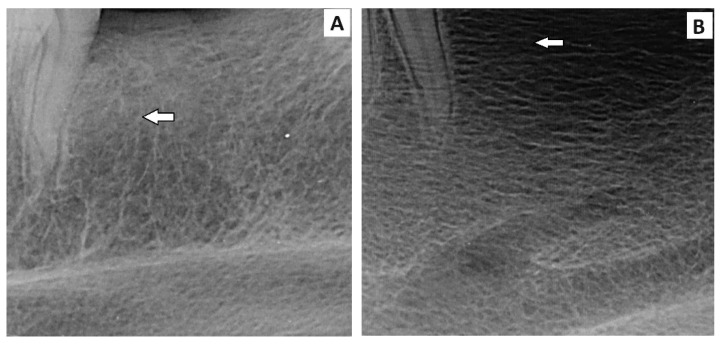
The intraoral X-ray examination. The white arrows show the site of the *experimental group* treated with CAS, (**A**) and the *control* sample (**B**) after 6 months healing.

The standard radiographic (X-ray) and computed tomography (CT) methods were used to evaluate the treated mandible bone defects. Using standard X-rays in the latero-lateral position of the head, the complete integration of the newly formed bone with the surrounding tissue was revealed (Figure 5A), in comparison to the spontaneous healing of the bone defects in the control group of animals (Figure 5B). In addition, intraoral X-ray examination at the level of the second premolar confirmed the process of regeneration and formation of new bone tissue after the implantation of CAS cement, as well as physiological values of bone density in the experimental group (Figure 6A). In the control group, the bone density in the defect site was lower than in adjacent origin bone (Figure 6B).

**Figure 7:**
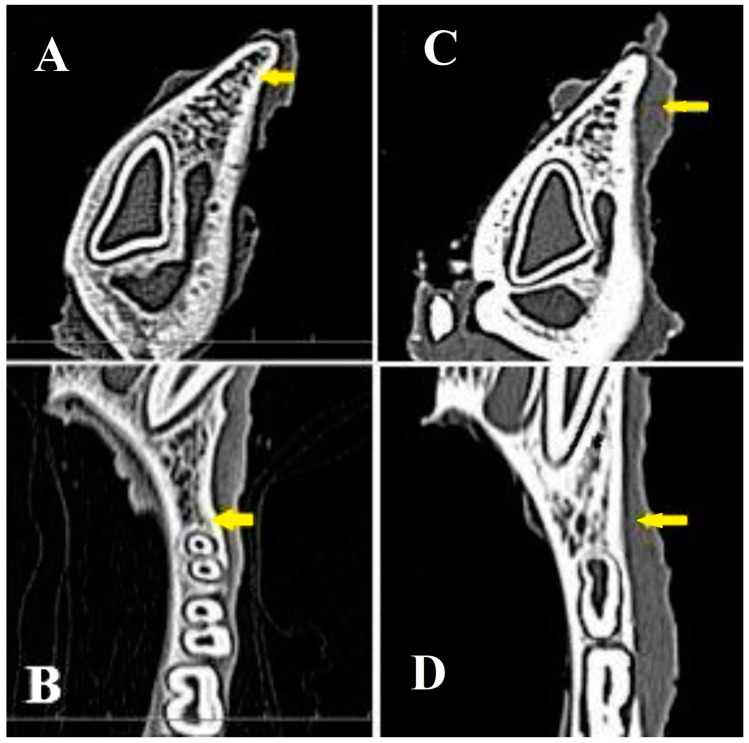
CT records of the axial plane (**A**), Pos: 0.3, Plane: (1.00, 0.00, −0.00, −0.28), W: 100, L: 35, and coronal plane (**B**) of bones and defect sites in the *experimental group* (treated with CAS), Pos: −21.3, Plane: (0.00, −0.00, 1.00, 21.25), W: 100, L: 35, which proves the total regeneration of the alveolar bone tissue. A partial regeneration of the bone in the area of the defect in the axial and coronal plane (**C**,**D**), was found in the *control group*. (yellow arrows show the surgical area).

The computed tomography in the axial and coronal planes verified alveolar bone reconstruction in the area of the implanted CAS biocement at the level of the second premolar. Moreover, the evaluation of healed mandible defects in the axial (Figure 7A) and coronal planes in the experimental group (Figure 7B) showed the complete integration of the newly formed bone tissue in full defect thickness without visible changes in the structure or integrity of the examined bones. Note that no bone resorption was observed in the defect site and new bone tissue thickness was almost identical to the adjacent origin bone. The computerized tomography demonstrates in both axial (Figure 7C) and coronal planes (Figure 7D) only partial regeneration of the bone defect in the control group.

## 4. Discussion

Calcium sulfate (CS) is utilized as a soluble non-cytotoxic porogen in calcium phosphate biocements [29] and is frequently used for improving the in vivo resorption of biocements and enhancing new bone tissue formation [30]. The addition of CS to HAP cement revealed the markedly improved in vivo properties of composite with a gradual bioresorption over 12 weeks after implantation in rat muscle [31]. The stimulatory potential of CS on the growth activity of bone cells was also studied in socket preservation surgery after tooth extraction. The CS was successfully applied in free form or enriched with platelet-rich plasma (PRP) as a post-extraction dental filling in five hybrids [32]. A strong vascular invasion was demonstrated in the alveolar ridge treated with CS in allograft paste in patients (61% of vital bone compared to 26% in the bovine xenograph group) [33]. Beuerlein et al. [34] described the long-time safe utilization of calcium sulfate as a bone substitute and documented medical studies that support the use of calcium sulfate as a bioabsorbable bone substitute. The favorable impact of calcium sulfate on alveolar ridge preservation in human patients was reported. Brkovic and Radilovic [35] reported an extraction of an endodontically treated first premolar. Immediately after the tooth extraction, calcium sulfate was applied into the bone defect. Seven months later, a solid alveolar bone with appropriate vertical and horizontal dimensions for implant placement was confirmed. The grafted material was replaced by a newly formed trabecular bone without inflammatory cells, but with numerous osteocytes in the lacunae. The results support the efficacy of calcium sulfate as a grafting material for ridge preservation prior to implant placement. Huang et al. [36] reported a case of a healthy female patient who received calcium sulfate as the sole grafting material for augmenting the alveolar ridge dimensions of the socket of tooth 16 over a 2.5-year follow-up period. Medical-grade calcium sulfate mixed with normal saline was placed into the tooth socket. The complete closure of the socket of tooth 16 was revealed 3–5 months after CS implantation. Finally, 2.5 years after the surgery, the operation site healed completely with a healthy edentulous area.

Kelly et al. [37] published a study with 109 human patients with bony defects (tumors, trauma, periprosthetic bone loss, etc.) which were treated with calcium sulfate pellets. CS pellets were found to be 90% resorbed 2 months after surgery and approached 100% resorption by the third month. The percentage of bone growth and the replacement of the calcium sulfate was 80% of the original defect size at 2 months and approached 100% 6 months post-surgery. The study showed evidence for the use of calcium sulfate bone substitute either alone or as an expander of other graft materials in the treatment of bone defects.

Two months after extracting the first premolar in eight healthy mongrel dogs, bilateral intrabony defects were created mesial to the mandibular second premolar. One side was grafted with nanocrystalline CS; the opposite side was grafted with microcrystalline CS. The histological changes were recorded, and histomorphometric and radiographic analysis was conducted at 1 month and 3 months post-surgery. The authors found that nanocrystalline calcium sulfate significantly enhanced periodontal regeneration compared to the microcrystalline form [38].

Numerous studies with calcium phosphates/calcium sulfate have been reported. A large study involving socket preservation through augmentation techniques in 454 patients treated with Bond Apatite readymade composite bone graft material (biphasic calcium sulfate/hydroxyapatite composite cement) demonstrated successful graft incorporation without an inflammatory response. Three months post augmentation, the new bone was identified to contain a small amount of residual composite particles and connective tissue [13]. It was concluded that pure biomimetic HA, nanocrystalline HA grafting, carbonated hydroxyapatite, or 3D-printed nano-porous hydroxyapatite materials could be applied into fresh extraction sockets to limit bone resorption [1,9,14]. Defects resulting from the first molar tooth extraction in the mandibles of dogs were treated with hydroxyapatite cement and no signs of inflammatory response or infection were detected [39]. Additionally, histological analysis revealed a high degree of variability in nanohydroxyapatite resorption and osteoconduction following the application of a nanocrystalline hydroxyapatite paste immediately after tooth extraction in the extraction socket of the second molar in dogs. Based on these findings, it was concluded that nanohydroxyapatite may not be suitable for socket preservation procedures, as it failed to prevent dimensional ridge alterations due to unpredictable material resorption. [40]. Contrary to the above facts, a novel nanohydroxyapatite/mineralized silk fibroin composite scaffold induced bone formation and so preserved the height of the alveolar ridge after tooth extraction [6]. Jaita et al. [41] synthesized and characterized a hydroxyapatite–calcium sulfate bone cement for bone repair. An in vivo histological analysis following implantation in rabbit femurs demonstrated new bone formation around defects. Osteoblasts were found peripherally on the bone trabeculae, and osteoblast-like cells were observed within the granules 4–8 weeks post implantation.

In the case of CAS cement, no remains of calcium phosphate particles from the cement were found after six months of healing and newly formed alveolar bone tissue was identified in the area without signs of inflammation. Additionally, no dimensional ridge abnormalities were measured and new bone tissue in the mandible had the same thickness, morphology, and density as adjacent bone. These results clearly demonstrate the appropriate stimulation effect of CAS in enhancing the bioactivity of CPC as well as the activity of cells for the bioresorption of cement phases and subsequent new bone tissue formation. In addition, more complicated composite scaffolds composed of, for example, biphasic calcium phosphate/collagen membrane are more commonly used after tooth extractions than simple CAS cement, but they ensure good bone and soft tissue properties and preserved ridge dimensions after the healing of the treated bed sockets following tooth extraction for future implant placement [16]. Bio-Oss^®^ (Geistlich Pharma AG, Wolhusen, Switzerland, a deproteinized bovine bone mineral) is frequently applied for filling extraction sockets, but it was found that in defects grafted with Bio-Oss the connective tissue and small amounts of new bone tissue surrounding the graft particles are formed after 7 months of healing [42]. On the other hand, histological examination of grafted sites with Bio-Oss in 15 patients after 9 months of healing demonstrated that Bio-Oss is a biocompatible material suitable for fresh extraction sockets and capable of preserving alveolar bone [43]. Although bone allografts represent the gold standard in bone reconstruction and regenerative medicine, the augmentation of sockets with demineralized freeze-dried bone allografts has often failed and very low levels of new bone formation have been reported [44,45].

CAS cement mixture represents a non-cytotoxic, bioresorbable system with a positive effect on socket preservation, as examined 6 months post-surgery in pigs. However, the study’s limitation is, of course, this relatively short time period. It is not possible to predict if the alveolar bone will maintain its dimensions in the coming months or years due to the missing tooth and the consequent absence of micromechanical stimuli at the mandibular site.

On the other hand, it would be interesting to include an examination of the resorption of CPC and CS particles as well as the condition of the implantation site in a shorter healing time, for example 3 months post-surgery. In addition, it would also be interesting to compare the healing processes after treatment with CAS and with CS alone as a control group.

Nevertheless, it is crucial to address the ethical issues involved in experiments with large mammals and animals in general. Additionally, the manipulation and examination of the health status of pigs can be relatively difficult compared to smaller laboratory animals. The healing results of tooth extraction sites using calcium phosphate biomaterials in the form of scaffolds or cement pastes are generally often contradictory, and the composition of the cement as well as other physico-chemical or morphological properties of the cement particles must be carefully considered in order to obtain optimal properties for achieving the desired stimulating effect in terms of bone growth after surgery and the preservation of the socket.

## 5. Conclusions

The tetracalcium phosphate/monetite/calcium sulfate hemihydrate powder cement mixture was applied to treat post-extraction tooth sockets in pigs. Six months post-surgery, macroscopic examination revealed alveolar bone tissue characterized by a fairly smooth and homogeneous surface at the defect site. The calcified alveolar bone exhibited a morphology, thickness, and bone density similar to that of the adjacent native bone, with no remaining biocement. Moreover, no inflammation was observed, indicating excellent biocompatibility and osteointegration, which suggests the potential clinical application of the biocement CAS in human socket preservation following tooth extraction.

It is important to note that the long-term stability of the treated area after healing cannot be predicted at this time. However, this question could be addressed in future experiments.

## Data Availability

Data is contained within the article.
